# Basic and Clinical Advances in the Diagnosis and Management of Migraine

**DOI:** 10.1155/2020/8958143

**Published:** 2020-03-12

**Authors:** Xiaolei Shi, Wei Di, Aneta Wieczorek

**Affiliations:** ^1^Yijishan Hospital, The First Affiliated Hospital of Wannan Medical College, Wuhu, China; ^2^Shaanxi Provincial People's Hospital, Third Affiliated Hospital of Medical College, Xi'an Jiaotong University, Xi'an, China; ^3^Institute of Dentistry, Jagiellonian University Medical College, Krakow, Poland

Migraine is a common headache disorder and is one of the highest causes of disability among the population in the world [1]. It is estimated that 20.2% of women and 9.4% of men suffer from this disorder [2]. It is typically unilateral and frequently present in the form of throbbing or pulsating sensation and other associated symptoms, including nausea, vomiting, phonophobia, and photophobia [3]. Usually, the symptoms may last several hours to days, severely impairing the quality of life. It is believed that neuroinflammation, dysfunction of the descending pain-modulating network, altered trigeminal and autonomic system function, and other mechanisms may contribute to migraine [4–6]. Moreover, recent studies have provided new findings in the genetic causes, anatomical and functional characteristics, and pathological potentials of migraine. For example, genomic loci associated with migraine were enriched in genes that are expressed in gastrointestinal tissues [7]. This may explain the gastrointestinal symptoms concomitant with pain attacks, like nausea, vomiting, and the like. Studies have suggested that migraine headache is significantly associated with infant colic and inflammatory bowel disease [8, 9].

This special issue aims to cover migraine-related studies and provide a multidisciplinary treatment strategy for it. In this issue, readers will find six papers studying a wide spectrum of aspects of migraine: “Human Urinary Kallidinogenase Reduces Lipopolysaccharide-Induced Neuroinflammation and Oxidative Stress in BV-2 Cells” by Z. Zhao et al., “Curcumin Protects Human Umbilical Vein Endothelial Cells against H_2_O_2_-Induced Cell Injury” by J. Ouyang, “Cognitive Decline in Chronic Migraine with Nonsteroid Anti-Inflammation Drug Overuse: A Cross-Sectional Study” by X. Cai et al., “Effects of Diet Based on IgG Elimination Combined with Probiotics on Migraine Plus Irritable Bowel Syndrome” by Y. Xie et al., “The Relationship between Infant Colic and Migraine as well as Tension-Type Headache: A Meta-Analysis” by D. Zhang et al., and “Effect of Core Stability Training Monitored by Rehabilitative Ultrasound Image and Surface Electromyogram in Local Core Muscles of Healthy People” by Y. Zheng et al.

The studies included in this issue will help understand and develop new research in a most recent viewpoint.

*Xiaolei Shi* *Wei Di* *Aneta Wieczorek*
